# In Vivo Antiphytoviral and Aphid Repellency Activity of Essential Oils and Hydrosols from *Mentha suaveolens* and *Foeniculum vulgare* to Control Zucchini Yellow Mosaic Virus and Its Vector *Aphis gossypii*

**DOI:** 10.3390/plants12051078

**Published:** 2023-02-28

**Authors:** Anna Taglienti, Livia Donati, Immacolata Dragone, Luca Ferretti, Andrea Gentili, Fabrizio Araniti, Filippo Sapienza, Roberta Astolfi, Simona Fiorentino, Valerio Vecchiarelli, Claudia Papalini, Rino Ragno, Sabrina Bertin

**Affiliations:** 1 Research Centre for Plant Protection and Certification, Council for Agricultural Research and Economics, 00156 Rome, Italy; 2 Department of Agricultural and Environmental Science, University of Milan, 20122 Milan, Italy; 3 Rome Center for Molecular Design, Department of Drug Chemistry and Technology, Sapienza University of Rome, 00185 Rome, Italy; 4 Centro Appenninico del Terminillo “Carlo Jucci”, Perugia University, 02100 Rieti, Italy; 5 ARSIAL Regional Agency for the Development and Innovation of Agriculture of Lazio, 00162 Rome, Italy

**Keywords:** essential oil, hydrosol, plant virus, aphid vector, antiphytoviral, repellency, defense response

## Abstract

In recent years, natural compounds have gained attention in many fields due to their wide-range biological activity. In particular, essential oils and their associated hydrosols are being screened to control plant pests, exerting antiviral, antimycotic and antiparasitic actions. They are more quickly and cheaply produced and are generally considered safer for the environment and non-target organisms than conventional pesticides. In this study, we report the evaluation of the biological activity of two essential oils and their corresponding hydrosols obtained from *Mentha suaveolens* and *Foeniculum vulgare* in the control of zucchini yellow mosaic virus and its vector, *Aphis gossypii*, in *Cucurbita pepo* plants. The control of the virus was ascertained with treatments applied either concurrently with or after virus infection; choice tests were performed to verify repellency activity against the aphid vector. The results indicated that treatments could decrease virus titer as measured using real-time RT-PCR, while the experiments on the vector showed that the compounds effectively repelled aphids. The extracts were also chemically characterized using gas chromatography–mass spectrometry. *Mentha suaveolens* and *Foeniculum vulgare* hydrosol extracts mainly comprised fenchone and decanenitrile, respectively, while essential oils analysis returned a more complex composition, as expected.

## 1. Introduction

Essential oils (EOs) are volatile, complex mixtures of strong-scented natural compounds synthesized through the specialized metabolism of aromatic plants typical of temperate and tropical regions and stored in secretory cells, glands or trichomes [1]. They are usually extracted by means of steam- or hydro-distillation (SD or HD) [2] and using liquid or supercritical carbon dioxide [3]. EO chemical composition is analyzed using gas chromatography–mass spectrometry (GC-MS); their main components are terpenes, terpenoids, aromatic and aliphatic low-molecular-weight molecules and their oxygenated derivatives. In nature, EOs are involved in plant defense systems as antibacterials, antivirals, antifungals, insecticides and repellents [4].

Because of their wide-range biological activity [5,6,7], EOs are used in the pharmaceutical and food industry and are increasingly popular as antimicrobials in plant protection. In all these sectors, the rising concern towards synthetic molecules regarding drug resistance, toxicity and environmental contamination has pushed researchers to test natural products as alternatives or adjuvants to classical synthetic remedies. EOs provide low residue levels and reduced risk of resistance development in target organisms, resulting in a safer profile with respect to conventional pesticides [8]. 

Hydrosols (HSs) are the by-products of plant SD during EO production. HSs contain small amounts of EO components solubilized in condensing water during the distillation process and are mainly composed of polar, oxygenated and hydrophilic compounds able to form hydrogen bonds [9]. As they are waste products, HSs are relatively cheaper and can be produced in higher amounts than EOs; moreover, they are reported to be less detrimental to human health.

Plant virus diseases represent a major concern for crops, causing considerable damage and economic losses worth USD 60 billion worldwide [10]. No direct application of compounds of either natural or synthetic origin is available for virus treatment in plants [11]; hence, control lies in prevention, e.g., the use of sanitarily certified propagation material and resistant plant varieties, and control of insect vectors [12,13]. Recently, the antiphytoviral activity of several EOs has been reported, mainly aiming at moderating symptomatology and yield loss. Experimental trials have often involved plant–virus model systems and have reported the inhibition of local lesions developed by virus infection due to EO treatment [14,15,16,17]. Later, Abdel-Shafi and co-authors investigated the potential activity of EOs in an actual pathosystem of agronomic interest, using *Nigella sativa* seed extract to effectively control zucchini yellow mosaic virus (ZYMV) in *Cucurbita pepo* both in vitro and in vivo [18]. The inhibition of cucumber mosaic virus (CMV) with associated satellite RNA upon treatment with *Micromeria croatica* EO in *Nicotiana megalosiphon* was ascertained, showing a decrease in virus concentration in systemically infected plants [19]. In a previous paper, we assessed the antiphytoviral activity of EOs and HSs from *Origanum vulgare*, *Thymus vulgaris* and *Rosmarinus officinalis* against ZYMV and tomato leaf curl New Delhi virus in *C. pepo* [20]. The antimicrobial activity of HSs has been reported, mainly in the post-harvest treatment of food products against biotic spoilage [21,22,23,24]; nonetheless, the application to plant viruses is still poorly described and understood, and more investigation is needed. The efficient delivery of compounds to plants for pathogen control is another key issue for the applicability of such treatments; formulations and smart delivery systems, which are mainly based on nanoparticles loaded with agrochemicals or other compounds, have been proven to effectively provide correct penetration and transport of the active molecule into plants, reducing damage to other plant tissues [25,26,27,28,29,30].

Arthropod vectors represent the other key target for controlling virus diseases worldwide. Currently, the main control strategy is based on synthetic insecticides, but the associated environmental and health risks, along with the development of resistant populations, make the search for suitable alternatives necessary. The insecticide, repellent and antifeedant activities of EOs against arthropods, including virus vectors, have been extensively studied and reviewed [31,32]. In addition, HSs have been successfully tested against hemipteran and mite vectors [33,34,35].

Among the aromatic plants used as a source of both EOs and HSs, the *Lamiaceae* and *Apiaceae* families have been shown to have several biological activities. In the Lamiaceae, both EOs and HSs extracted from *Mentha* spp. have been well characterized and reported to have antiviral activity as well as toxic and repellent effects on insects [34,36]. In the Apiaceae, *Foeniculum vulgare* provides EOs that have broad activity against several plant pathogenic bacteria and fungi [37,38,39] and have been reported to have insecticide efficacy against aphid vector *Myzus persicae* [40]. Based on these findings, we hypothesized that EOs and HSs extracted from *Mentha suaveolens* and *F. vulgare* could effectively control ZYMV and its vector, *A. gossypii*, in *C. pepo*. ZYMV (genus *Potyvirus*, family *Potyviridae*) is an ssRNA (+) plant virus mainly infecting Cucurbitaceae, including major crops such as pumpkin, squash, zucchini, melon, watermelon and cucumber [41]. The observed symptomatology is severe mosaic and yellowing of leaves; stunting, twisting and deformation occur in fruits, negatively affecting their marketability. After infection, plants no longer provide marketable production within 1–2 weeks. Such a rapid settlement of severe disease causes a yield decrease of up to 90% and subsequent economic losses [42]. The use of resistant varieties is currently a valid strategy for the control of ZYMV in *C. pepo*, but resistance-breaking strains are increasingly widespread; hence, resistance should always be used in combination with other control methods [43]. ZYMV is transmitted by several aphid species in a non-persistent manner. *Aphis gossypii* Glover (Hemiptera: Aphididae) plays a key role in ZYMV spread because it is widely distributed worldwide and has a high transmission efficiency [44]. Moreover, it has been reported to rapidly develop resistance to several synthetic insecticides [45].

Given the above-described data and hypothesis, and the urgent need for natural compounds to treat plant diseases and pests, we investigated the antiphytoviral and aphid-repellent activities of either EOs or HSs extracted from *M. suaveolens* and *F. vulgare*. Plants were treated with EOs and HSs concurrently with virus inoculum to measure the EO or HS effect on virus vitality and infectivity in vitro, and after virus inoculation, to test the “curative” activity of extracts. The potential effects against ZYMV were assessed by comparing the relative virus titer as measured with real-time RT-PCR in systemically infected leaves of treated and untreated plants of *C. pepo*. Moreover, the expression of the phenylalanine ammonia lyase (PAL) gene was quantified to explore a possible mode of action for treatments in plants. PAL is the first enzyme of the phenylpropanoid pathway, which is involved in plant defense mechanisms; the expression of the related gene is reported to be a marker of plant response to biotic stress through transcriptional regulation [46,47]. Choice tests of *A. gossypii* specimens between treated and untreated leaves were also performed to measure the repellent action of EOs and HSs against the ZYMV vector; the toxicity of treatments was also evaluated by measuring mortality and fecundity in adults. Our results showed that the tested extracts displayed antiphytoviral and repellency activity against the target virus and vector.

## 2. Results

### 2.1. Chemical Composition of Plant Extracts

The chemical classes identified using GC-MS in EOs and HSs are summarized in Table 1, while the detailed chemical composition is provided in Table S1. A total of 60 molecules belonging to 9 classes of compounds and 19 molecules belonging to 6 classes were identified in EOs and HSs, respectively; thus, EOs generally had a more complex composition than HSs. The identified compounds accounted for 82.38–94.75% of EO or HS composition. Monoterpene hydrocarbons and oxygenated monoterpenes were the main components of EOs; HSs were generally rich in oxygenated monoterpenes. The *M. suaveolens* EO composition was variegated, with 42 identified compounds showing relative abundance above 0.1%; the main compounds were isomintlactone and menthene (7.72 and 7.28%, respectively). Monoterpene hydrocarbons and oxygenated monoterpenes accounted for about 40% of the composition, followed by minor amounts of esters (about 10%). In the corresponding HS, the main component of the ether extract was identified to be decanenitrile (68.46%), which was not recognized in *M. suaveolens* EO. Oxygenated monoterpenes were also present in fair abundance (about 16%). Regarding *F. vulgare* extracts, EO contained monoterpene hydrocarbons and oxygenated monoterpenes for about 60%, while esters and aromatic compounds accounted for about 30%. Its HS displayed the main component of the extract, i.e., fenchone, at 75.93% relative abundance; it was also present in the corresponding EO but at a lower concentration (16.63%). 

### 2.2. Effect of Plant Extracts on Virus Titer in ZYMV-Infected Plants

#### 2.2.1. Treatments Applied at the Same Time as Inoculation

**Figure 1:**
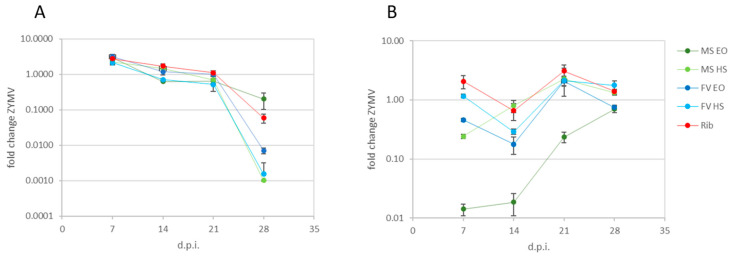
Fold changes of ZYMV in plants treated at the same time as inoculation (**A**) and treated after inoculation of ZYMV (**B**) on leaves harvested 7, 14, 21 and 28 d.p.i. MS EO = *M. suaveolens* essential oil; MS HS = *M. suaveolens* hydrosol; FV EO = *F. vulgare* essential oil; FV HS = *F. vulgare* hydrosol; Rib= ribavirin. Values are expressed as means of 2 technical replicates on 3 pooled biological replicates, and bars indicate standard error (±SE).

The evolution of the ZYMV relative titer in leaves of treated *C. pepo* plants over time was investigated with repeated weekly sampling between 7 and 28 days post infection (d.p.i.) as described in Section 4.4.1. In Figure 1, panel A reports the fold change (i.e., 2^−ΔΔCt^) values of the relative ZYMV titer obtained at the four sampling times with all treatments. In the first sampling instance, 7 d.p.i., the results showed a 2–3-fold increase in the virus relative titer with all treatments, including ribavirin. However, 14 d.p.i., the fold change of relative virus titer was around zero with all treatments, indicating no effects of applying EOs nor HSs on the control of ZYMV. Then, 21 d.p.i, all EO and HS treatments consistently had fold changes of the order of magnitude of 10^−1^, suggesting an effect of treatments on reducing the relative virus titer. Finally, 28 d.p.i., a major influence of treatments was noticeable on the relative virus titer; with *M. suaveolens* HS and *F. vulgare* extracts, fold changes of the order of magnitude of 10^−3^ were observed, while the relative virus titers were decreased by 10^−1^ in *M. suaveolens* EO-treated plants and by 10^−2^ in ribavirin-treated plants. In samples of all mock-inoculated plants in this and the following sections, the virus was not detected at any time, and such samples were not included in the graphs. Symptoms of phytotoxicity were not observed in any of the treated plants in this and the following sections, with all EOs and HSs.

#### 2.2.2. Treatments Applied after Inoculation

In Figure 1, panel B shows the evolution of the ZYMV relative titer over time when treatments were applied after inoculation. An early decrease in relative virus titer was observed 7 d.p.i. in treatments with *M. suaveolens* extracts of the orders of magnitude of 10^−2^ with EO and 10^−1^ with HS. With *F. vulgare* EO, the virus titer was around one half of the infected untreated control, while no changes were recorded with *F. vulgare* HS treatment. Two weeks after inoculation, *M. suaveolens* extracts maintained the same levels of virus titer observed in the first sampling instance, while *F. vulgare* extracts showed a decrease, reaching one-tenth of the control with EO and one-third with HS. Only *M. suaveolens* EO-treated plants showed a relative virus titer below the control 21 d.p.i., while all other treatments were 2–3-fold higher. In the final sampling instance, 28 d.p.i., both EOs displayed a virus titer decrease of the order of magnitude of 10^−1^, while in HS treatments, the virus was at levels above the control. The positive-control treatment with ribavirin had the same trend as most experimental treatments, showing a peak decrease in virus titer 14 d.p.i. 

### 2.3. Effect of Plant Extracts on Phenylalanine Ammonia Lyase Gene Expression

#### 2.3.1. Treatments Applied at the Same Time as Inoculation

**Figure 2:**
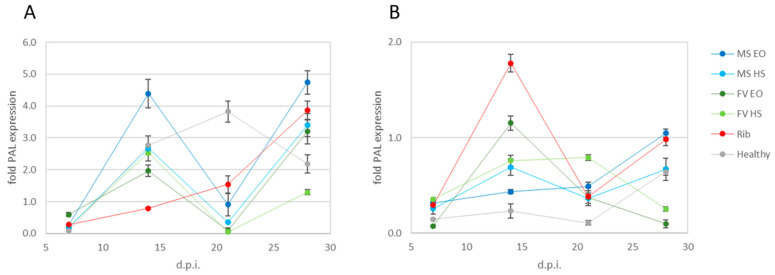
Relative expression of PAL in plants treated at the same time as inoculation (**A**) and treated after inoculation of ZYMV (**B**) in leaves harvested 7, 14, 21 and 28 d.p.i. MS EO = *M. suaveolens* essential oil; MS HS = *M. suaveolens* hydrosol; FV EO = *F. vulgare* essential oil; FV HS = *F. vulgare* hydrosol; Rib = ribavirin; Healthy = mock-inoculated plants. Values are expressed as means of 2 technical replicates on 3 pooled biological replicates, and bars indicate standard error (±SE).

The evolution of PAL expression (i.e., 2^−ΔΔCt^) in plants when treatments were applied at the same time as inoculation is shown in Figure 2, panel A. Initially, 7 d.p.i., all treatments, including ribavirin and healthy plants (i.e., mock-inoculated plants), showed underexpression of PAL (fold change of 0.08–0.59) compared with the infected untreated control. In the second observation, 14 d.p.i., PAL was overexpressed in all treatments, with fold changes between 1.96 with *M. suaveolens* HS and 4.39 with *F. vulgare* EO. In ribavirin-treated plants, PAL was still slightly underexpressed compared with infected untreated plants (fold change of 0.79). All treatments caused underexpression of PAL 21 d.p.i., of the orders of magnitude of 10^−2^ with *M. suaveolens* extracts and 10^−1^ with *F. vulgare* extracts, while ribavirin and healthy groups showed overexpression of PAL. At the final sampling time, 28 d.p.i., all treatments consistently overexpressed PAL, with fold changes between 1.29 and 4.74. As observed 14 d.p.i., for *F. vulgare* EO we recorded the highest level and for *M. suaveolens* HS the lowest level of overexpression.

#### 2.3.2. Treatments Applied after Inoculation

PAL relative expression in plants treated after inoculation is shown in Figure 2, panel B. Overall, no significant changes in the expression levels of the gene were observed with any experimental treatment at any sampling time. Ribavirin-treated plants had a peak PAL expression 14 d.p.i.

### 2.4. Effect of Plant Extracts on Virus Vector Choice and Survival 

Both EOs and HSs obtained from *M. suaveolens* and *F. vulgare* were tested to determine their potential effects on the settling of *A. gossypii* adults on treated *C. pepo* leaves, and adult survival and fecundity. All the bioassays were performed using 300 μg/mL EO or 1:2 *v*/*v* HS concentration, which were the same used for plant treatments (Section 4.4.1 and Section 4.4.2).

#### 2.4.1. Repellency

**Figure 3:**
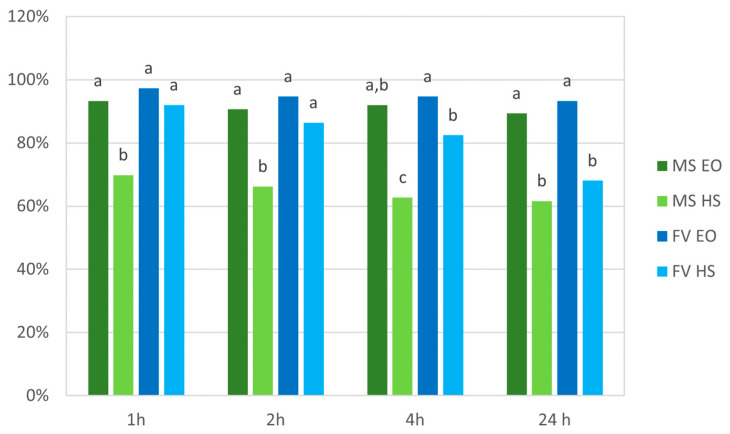
Test choice repellency assay using *M. suaveolens* and *F. vulgare* essential oils and hydrosols against *A. gossypii* adults. Twenty replicates were carried out, and 10 adults were used per replicate (N = 200). MS EO = *M. suaveolens* essential oil; MS HS = *M. suaveolens* hydrosol; FV EO = *F. vulgare* essential oil; FV HS = *F. vulgare* hydrosol. Different letters indicate significant differences among treatments at the same time of observation, based on Chi-square test.

Choice tests between EO- or HS-treated and water-treated leaves showed that a significantly higher proportion of aphids preferred to settle onto the control leaves rather than the treated leaves with all the tested compounds (Figure 3). This repellency effect started 1 h after treatment and persisted for 24 h. The repellency ascribable to *M. suaveolens* was significantly stronger with EO than with HS in all the time intervals. *F. vulgare* EO and HS had similar effects on aphid settlement 1 h and 2 h after treatment; then, HS repellency significantly decreased at 4 h and 24 h. In general, *M. suaveolens* HS was less efficient than the other compounds in inhibiting *A. gossypii* adults from settling on treated leaves. 

#### 2.4.2. Toxicity and Fecundity

Both the *M. suaveolens* and *F. vulgare* HS treatments significantly increased the mortality rates of *A. gossypii* adults compared with the control water treatment in most of the observed time intervals (Table 2). *M. suaveolens* HS was responsible for a highly significant toxic effect early after treatment, causing 58.6 and 62.1% of mortality after 24 and 48 h, respectively. The survival of *F. vulgare* HS-treated aphids was significantly reduced 24, 72 and 96 h after treatment compared with the control, but this effect was not as sharp as the *M. suaveolens* HS effect. Treatments with *M. suaveolens* and *F. vulgare* EOs showed lower levels of aphid mortality, which often did not significantly differ from the water control, especially in the earliest daily data collection instances. 

*M. suaveolens* HS treatment was also responsible for significantly reducing offspring production compared with the water control in the first three daily data collection instances (Table 3). No significant differences in aphid fecundity were found between the other treatments and the control.

## 3. Discussion

The use of natural resources from plant species to control plant viral diseases and their insect vectors has been broadly investigated in recent years. In particular, EOs and HSs have gained interest due to the important role they play in nature in the protection of plants as antibacterials, antivirals, antifungals, insecticides and repellents of undesirable insects. A treatment based on a natural compound with safety characteristics favorable for human health and the environment, and effective in controlling plant viruses and repelling their insect vectors is highly necessary for modern agricultural systems worldwide. EOs and HSs have been tested as antiphytovirals, insecticides and repellents, reporting promising results [31,48,49,50]. During our previous study, we investigated the potential biological activity of essential oils and hydrosols from *O. vulgare*, *T. vulgaris* and *R. officinalis* against ZYMV and tomato leaf curl New Delhi virus (ToLCNDV) in *C. pepo* [20]. The results of our study supported the feasibility of using such compounds to control ToLCNDV, whereas poor biological activity was observed against ZYMV. The established experimental protocol, involving inoculation, treatment, sampling, and measurement of virus titer and plant gene expression, was considered reliable and reproducible for further use in similar studies. Hence, in this study, we used the same system to test more EOs and HSs from two other plant species, *M. suaveolens* and *F. vulgare*; we also report their repellent activity against the aphid vector of ZYMV, *A. gossypii*, to potentially combine control of virus and vector with the same treatment. The choice of the two plant sources of extracts was based on the repellency activity against *A. gossypii* exerted by *M. suaveolens* and *F. vulgare* extracts, which was ascertained in a preliminary broader screening involving different extracts from five plant species. Given the above-mentioned activity, we hypothesized that *M. suaveolens* and *F. vulgare* EOs and HSs could also effectively control ZYMV in *C. pepo*; the extracts were first characterized using GC-MS and then tested as treatments on ZYMV-infected *C. pepo* plants in vivo. The verification of our hypothesis was accomplished by means of (i) the measurement of the relative virus titer in new leaves of systemically infected, treated plants and (ii) the evaluation of the relative expression of the PAL gene, to investigate a potential mechanism of action of the extracts. In fact, the biological activity of natural compounds is often ascribed to an indirect mode; rather than directly damaging the target organism, they are assumed to stimulate plant defense response, reducing pathogen load and symptomatology, making the host more tolerant to the pathogen, and ultimately remaining productive [32].

A comprehensive evaluation of treatments with natural compounds necessarily implies the chemical characterization of extracts, which can present broad variability of composition due to many factors related to the plant material (harvesting season, plant chemotype and cultivar, type of tissue and age of the plant) and the distillation method [51]. Hence, it is crucial to characterize the actual composition of the extract used in treatments, to know the compounds applied to the plant and possibly to identify biologically active molecules in the mixture. Given the variability of the extract composition, the knowledge of active molecules is, therefore, of great importance; under certain restraints, the actual performance of an extract can be assessed by verifying the presence of active molecules, whatever the residual composition.

EO and HS dissolved organic compounds were chemically characterized using GC-MS in terms of qualitative and quantitative relative composition. *M. suaveolens* EOs from different sources and geographical origins were already studied in the literature [52,53], and overall, all showed high percentages of oxides, which is confirmed by our results. Decanenitrile, the main compound of *M. suaveolens* HS, was not reported in high concentration in previous literature studies on this extract; the significant presence of hydrophilic oxygenated compounds previously observed, which is a typical feature of HSs, is instead in accordance with our results. Regarding *F. vulgare* extracts, the fairly high concentrations of fenchone and anethole observed in EO were already reported in the literature [54], whereas pentanedioic acid (p-t-butylphenyl)ester was found in high concentration in our experiments but was not previously reported in fennel extracts. As expected, *F. vulgare* HS ether extract was enriched in fenchone, an oxygenated compound already present in EO and accumulating in HS due to its polar moiety. 

The characterization of both *M. suaveolens* HS and *F. vulgare* EO thus evidenced the presence in high concentrations of a compound not previously reported in similar extracts. This is quite common in the study of plant extracts, whose composition is strongly affected by the numerous factors mentioned above and may vary significantly. 

The experiments in which treatments were mixed with virus inoculum and then applied to the plants (described in Section 4.4.1) were aimed to first assess the potential effect of EOs or HSs on the vitality and infectivity of ZYMV in vitro. This procedure, well reported in the literature as “inhibition activity” assay, is often performed as a preliminary experiment to test the possible interaction of EOs or HSs and the virus under the simplest conditions, involving in vitro contact between virus and treatment; then, the effect on a plant inoculated with such mixture is observed [15,16,18,19]. This procedure is necessary, because viruses are obligate parasites, so the mere in vitro inhibition test is not feasible. In this situation, the mechanism of action of the treatment could be retrieved via interference with coat proteins or the inhibition of the formation of capsid proteins, which are necessary for adsorption or entry into the host plant.

In this assay, a late but effective response was observed in terms of a decrease in viral titer. Fold change was substantially insignificant until 21 d.p.i. with all treatments, whereas 28 d.p.i., a huge decrease was observed, outperforming ribavirin in the case of both *M. suaveolens* HS and *F. vulgare* extracts. All these extracts have been previously described for their biological activity [36,55,56,57], but they have never been tested for the control of phytopathological viruses. Menthene, carvone, limonene and eucalyptol have been described to be foremost responsible for most biological activities and were also detected in our extracts.

The experiments in which treatments were applied after virus inoculation were performed to assess the potential of treatments to reduce the damage of an established infection, i.e., “curative” activity, as it is referred to in the literature [16,18]. The evolution of the ZYMV titer with time was very different when treatments were applied after inoculation; this timing, even though in the frame of an experimental trial under controlled conditions, is probably more adherent to the actual situation of infection/treatment in crop management. In this experiment, the response to treatment was recorded earlier but to a smaller extent. The most effective treatment was *M. suaveolens* EO, which maintained its activity at all sampling times.

The evaluation of PAL expression levels in treated plants was performed to investigate the likely mode of action of treatments involving plant defense response; in fact, PAL is a key enzyme in the phenylpropanoid biosynthetic pathway, and such compounds play a role in plant defense against many pathogens [58]. PAL is upregulated upon virus infection [47,59] and is involved in resistance development [46]. In this work, the PAL gene was chosen to first assess the hypothesis of an indirect mode of action of treatments, i.e., stimulating plant response rather than directly damaging the target pathogen. The overexpression of the PAL gene upon treatment indicates a plant response activating metabolic pathways to produce defense compounds.

In treatments applied concurrently with inoculation, the upregulation of PAL was observed to have a bimodal trend with most treatments, with two peaks 14 and 28 d.p.i., whereas ribavirin had a consistently increasing trend resembling an exponential curve. The peak 14 d.p.i. was also confirmed when treatments were applied after inoculation for most extracts, including ribavirin. In our previous work, the expression of PAL was also measured in a similar experiment with EOs and HSs from other plant species; in such a trial, HSs generally displayed better performance than EOs, while for *M. suaveolens* and *F. vulgare*, a similar trend was not confirmed. Based on these results, we can speculate that the activity of PAL regulation is probably more ascribable to the chemical composition of single extracts than to the nature of the extract (EO or HS). 

PAL upregulation was already observed upon treatment with EOs; when these extracts were applied in the post-harvest treatment of fruits and vegetables to control molds, overexpression of PAL occurred in the treated material [60].

Another option for assessing the activity of plant treatments is the application before virus inoculation, i.e., “protective” activity, as referred to in the literature [16,19]; in such assays, the ability of treatments to prevent the establishment of systemic infection is evaluated. We did not perform this experiment, but to date, this application mode has been successfully tested on different pathosystems.

Finally, EO and HS treatments also showed promising repellent activity against the vector of ZYMV, *A. gossypii*. Many EOs are currently used as repellents against harmful insects, and they are consistently considered good alternatives to synthetic molecules; some of them are registered by environmental protection agencies for such use. This field is still extensively studied [31] and has been recently extended to the use of hydrosols for the same purposes [34,61]. Extracts from several species of the genus *Mentha*, such as EOs from *Mentha piperita* and *Mentha longifolia*, and HSs from *Mentha pulegium*, are known to actively repel aphids [34,62]. Our data showed that *M. suaveolens* extracts could also play an important role in inhibiting *A. gossypii* settlement onto treated plants, and especially, EOs repelled around 90% of the tested adults for at least 24 h after treatment. Regarding *F. vulgare* extracts, few data are currently available on their potential repellent activity [63]. Our results show that both HSs and especially EOs were able to efficiently reduce the settling of *A. gossypii* on treated zucchini leaves compared with the untreated control. As expected, the *F. vulgare* extracts contained high concentrations of fenchone and anethol. These compounds have already been targeted as bioinsecticides against aphids [40,64,65] and are now also strongly suggested for their potential role as repellents. 

*M. suaveolens* and *F. vulgare* EOs did not show significant toxic effects against *A. gossypii* adults at the applied concentrations. HSs induced higher mortality levels than the control at different time points, but only *M. suaveolens* HS showed to be highly effective within a short time after treatment. Indeed, this compound halved the aphid population in just one day and significantly reduced the offspring production of the remaining adult specimens. The promptness of the toxic effect is an essential requisite of aphicide compounds, which should reduce the target population before the aphids start to actively suck the host’s sap. In this way, the chance of virus acquisition/transmission, which naturally occurs through rapid sucking punctures, would decrease. The early toxic effect of *M. suaveolens* HS has already been assessed against another aphid species, *Toxoptera aurantii*, confirming the potential of this hydrosol in aphid control [35].

## 4. Materials and Methods

### 4.1. Aromatic Plants

The source plants were obtained through an ongoing project aimed to investigate how EO production and associated chemical composition can be influenced by different cropping techniques; in the frame of this project, *M. suaveolens* and *F. vulgare* plants were harvested and subjected to EO distillation and HS separation. *M. suaveolens* Ehrh and *F. vulgare* Mill. plants were grown at Stazione di Base del Centro Appenninico del Terminillo “Carlo Jucci” in Rieti (Italy). The initial transplant was performed in September 2016. The plants were harvested in summer 2018; then, they were dried, sealed and stored in a closet.

### 4.2. Essential Oil and Hydrosol Production and Characterization

#### 4.2.1. Essential Oils

EOs extracted in the frame of the project were obtained in low amounts to be used for all activities; hence, additional amounts were purchased from Farmalabor srl (Assago, Italy), and their chemical composition was analyzed using GC-MS (see Section 4.2.3).

#### 4.2.2. Hydrosol Preparation

*M. suaveolens* and *F. vulgare* plants were subjected to 2 h HD extraction of aerial parts using a Clevenger-type apparatus as previously described [66]. EOs were separated from HSs and stored in tightly closed dark vials at −18 °C until further utilization in other studies. Extractions were performed according to the protocol of European Pharmacopeia. Fresh leaves (2 kg) of aerial parts from each plant species were used for distillation. HSs were separated from EOs using decantation, avoiding the carryover of EOs. The HS organic part was extracted twice with diethyl ether (Sigma-Aldrich, Milan, Italy) in a separation funnel to eliminate water and was stored at 4 °C in brown glass vials in the dark until further analysis or testing. The EO/diethyl ether phase was dried over anhydrous sodium sulfate (Sigma-Aldrich, Milan, Italy), and diethyl ether was then evaporated.

#### 4.2.3. Gas Chromatography–Mass Spectrometry Analysis

GC-MS analyses of EOs and HSs were performed using an Agilent Technologies gas chromatograph (GC 7890A) coupled with a single quadrupole mass spectrometer (5975C Inert XL MSD) and an autosampler (CTC analytics PAL system). A 5MS (30 m × 0.25 mm × 0.25 µm + 10 m of pre-column; MEGA srl, Milan, Italy) column was used for sample chromatography, and ultra-pure helium (6.0 BIP; SAPIO srl, Monza, Italy) at a flow rate of 1 mL/min was used as the gas carrier. Injector, source and transfer line were settled at 280 °C, 280 °C and 250 °C, respectively.

Before injection, EOs were diluted to 1:10 *v*/*v* with n-hexane (97% purity; Sigma Aldrich, Milan, Italy), while HSs were previously liquid–liquid-extracted in n-hexane at 1:1 *v/v*. The organic fraction was separated, dried with anhydrous sodium sulfate, concentrated and directly injected. EOs were injected in split mode (split ratio of 1:50) at 50 mL/min split flow, whereas HSs were injected in splitless mode.

Sample separation was achieved using the following temperature ramp: 5 min at 60 °C; from 60 °C to 220 °C at a rate of 4 °C/min; from 220 °C to 280 °C at a rate of 11 °C/min; isocratic for 15 min 280 °C; then, from 280 °C to 300 °C at a rate of 11 °C/min.

Mass spectra were recorded in electronic impact (EI) mode at 70 eV. The analysis was conducted in full scan mode, from 30 to 450 m/z, with a solvent delay of 5 min. The retention index (RI) was calculated using a separately injected n-alkane standard mixture (C8-C30; Sigma Aldrich, Milan, Italy). 

The samples were injected three times, and the obtained chromatograms were aligned and deconvoluted using the open-source software MS-DIAL 4.8 [67]. The area of each compound was extracted and mediated. Peak annotation was achieved using the RI and spectral similarity matching with a cosine score cut-off of 70% using an in-house EI spectral library [68], following Metabolomics Standards Initiative of the International Metabolomics Society. In particular, as suggested by [69], the annotations were considered at level 2 (putative annotation based on spectral library similarity) or level 3 (putatively characterized compound class based on spectral similarity to known compounds of a chemical class). Moreover, the relative quantitation of these compounds was also calculated as the mean of the relative percentage for each peak (peak area/total ion current (TIC) area) over the three replicate injections (Table 1 and Table S1).

### 4.3. Experimental Plant Material

#### 4.3.1. Plant Host

Seeds of *C. pepo* ”Tullio” were sown in 12 cm plastic pots with “Completo” soil (Vigorplant, Italy) and germinated in a greenhouse (23 °C, 16:8 hr (L:D) photoperiod). The obtained plants were grown in an insect-proof greenhouse under the above-mentioned conditions. Experimental plants were selected 3 weeks after sowing when they had two fully expanded cotyledons. The selection was performed to ensure that the experimental plants were as uniform in size as possible. 

#### 4.3.2. Virus Inoculum

ZYMV isolate 31 from the Research Centre for Plant Protection and Certification (CREA-DC) collection was propagated in the plant host, *C. pepo* ”Tullio”, under the above-mentioned greenhouse conditions. Systemically infected young leaves were ground with cold 0.1 M pH 7.4 phosphate buffer (1:5 w/V) in an extraction bag (Bioreba, Switzerland) to prepare the virus inoculum for the antiphytoviral activity experiments. 

### 4.4. Experimental Trials

#### 4.4.1. Treatments Applied at the Same Time as Inoculation

The ZYMV inoculum was mixed with EO or HS solution in 0.1 M pH 7.4 phosphate buffer to obtain final concentrations of 1:10 *v*/*v* virus inoculum, 300 μg/mL EO or 1:2 *v*/*v* HS and incubated on ice for 1 h. Then, host plants in the developmental stage of fully expanded cotyledons were mechanically inoculated with the above-described solution of inoculum + treatment, and 20 μL was smeared on each cotyledon. Each treatment was applied on three biological replicates (i.e., plants), and the following controls were also included: (i) ZYMV-infected treatment with ribavirin as positive control (final concentrations: 1:10 *v*/*v* virus inoculum and 300 μg/mL ribavirin); (ii) ZYMV-infected non-treatment as negative control (final concentration: 10:10 *v*/*v* virus inoculum); and (iii) healthy control (mock-inoculated with phosphate buffer). Treatments were only applied once. When the first leaf was expanded, 7 d.p.i., plants were sampled by removing a disk from the above-mentioned leaf. More sampling was performed with the same procedure 14, 21 and 28 d.p.i. on the second, third and fourth expanded leaves, respectively.

Biological replicates (3 plants per treatment) were pooled, and the pools were analyzed as a single sample. Quantification of virus titer and expression of the PAL gene using real-time RT-PCR were performed as described below. All the experimental procedures reported in the present and the following sections are depicted as a flow diagram in Supplementary Figure S1.

#### 4.4.2. Treatments Applied after Inoculation

Host plants were inoculated under the same conditions described in the previous section but with no treatment added to the inoculum (final concentration: 1:10 *v*/*v* virus inoculum). After 5 h, treatments of EOs or HSs were applied to the inoculated cotyledons under the same conditions described in the previous section (final concentration: 300 μg/mL EO or 1:2 *v*/*v* HS). Application was performed by smearing inoculated leaves with EO or HS solution; treatments were only applied once. Biological replicates, control treatments and sampling procedures were the same as those described above. 

#### 4.4.3. RNA Extraction and Real-Time RT-PCR

Total RNA was extracted from collected samples using RNeasy Plant Mini Kit (Qiagen, Milan, Italy) according to the manufacturer’s instructions. Extracts were checked for purity and concentration with a NanoDrop™ spectrophotometer (ThermoFisher Scientific, Milan, Italy). Up to 10 μg of RNA was exposed to 2 U TURBO DNase™ (TURBO DNA-*free*™ kit; Life Technologies, Milan, Italy) in 10X TURBO DNase™ Buffer (total reaction volume of 50 μL) at 37 °C for 25 min. Then, 5 μL of DNase Inactivation Reagent from the kit was added; the mix was incubated at room temperature for 5 min to stop the reaction and centrifuged at 10,000 g for 90 s; then, the supernatant was recovered as DNA-depleted RNA for downstream analyses. 

TaqMan^®^ real-time RT-PCR assay was used for the relative quantification of ZYMV using primers and probe targeting the ZYMV coat protein (CP) gene [70]. The amplification reaction had a final volume of 20 μL, containing 2X TaqMan^®^ RT-PCR Master Mix, 40X TaqMan^®^ RT Enzyme Mix (TaqMan^®^ RNA-to-C_T_ 1-Step Kit; Life Technologies), 300 nM of each primer, 50 nM probe, and 1 μL of DNA-depleted RNA. 

The relative expression of PAL was analyzed with SYBR Green^®^ real-time PCR assay; first, RNA was reverse-transcribed to cDNA to convert plant transcriptome into DNA substrate for real-time PCR. The reaction had a final volume of 20 μL, containing 5X first-strand buffer (Invitrogen, Milan, Italy), 5 μM random hexamers (Promega, San Diego, CA, USA), 10 μM dNTPs (Promega), 100 U M-MLV (Promega) and 2 μL of RNA. The reaction was incubated for 45 min at 42 °C and 3 min at 94 °C in CFX96 Touch PCR System (Bio-Rad, Milan, Italy); then, 1 μL of RNase cocktail mix (Life Technologies) was added to remove traces of unreacted RNA. SYBR Green^®^ real-time PCR assay for PAL expression was performed using primers designed by Zhang et al. [71]. The reaction had a final volume of 10 μL, containing 2X SsoAdvanced™ Universal SYBR Green^®^ Supermix (Bio-Rad, Milan, Italy), 150 nM of each primer and 1 μL of cDNA template. All real-time (RT-)PCR assays were performed using CFX96 Touch RT-PCR System (Bio-Rad, Milan, Italy) with primers and probes synthesized by Eurofins Genomics (Ebersberg, Germany). The instrument automatically set the threshold. The virus relative titer and PAL expression levels were calculated using the method of ΔΔCt [72]. ZYMV-infected plants not exposed to treatments as described in Section 4.4.1 were considered the control group; the housekeeping gene was the elongation factor EF-1α gene of *C. pepo*, targeted with previously published primers (for SYBR Green^®^ assay) and probe (for TaqMan^®^ assay) [73]. All samples represented a pool of three biological replicates (i.e., plants subjected to the same treatment in the same pot) and were assayed in two technical replicates. Relative virus titers and PAL expression levels, defined as 2^−ΔΔCt^, were calculated with CFX Maestro Software ver. 2.2 (Bio-Rad, Milan, Italy), and the results were expressed as means ± standard error (SE).

#### 4.4.4. Insect Bioassays

A clonal colony of *A. gossypii* was reared on *C. pepo* ”Tullio” plants in insect cages (nylon net, 150 × 150 mesh) and maintained under controlled conditions at 25 ± 1 °C, 65 ± 5% RH and 16:8 hr (L:D) photoperiod.

Choice tests to investigate potential EO and HS repellency effects were carried out in 15 cm Ø Petri dishes, filled with moistened filter paper. In each dish, a zucchini leaf sprayed with EO or HS using a hand sprayer and a leaf of the same age and size sprayed with deionized water were placed onto filter paper with their lower surface facing upwards. The two leaves were separated with wet cotton to avoid possible interference between them, and the left/right position of the two leaves within the dish was inverted among the experimental replicates. An area of the dish equally far from the two leaves was kept free to host the aphids. Ten apterous adults of *A. gossypii* were transferred from the rearing cage to the free area of each dish using a fine brush and were allowed to spread and start sucking on the leaves. Petri dishes were then sealed with parafilm. The number of aphids that settled on each leaf was recorded 1, 2, 4 and 24 h after treatment. Twenty replicates were performed for each EO or HS treatment. Repellency was calculated based on the counts of adults on untreated and treated leaves using the following equation [62]:

$$R\left(\%\right)=\frac{A\left(untreated\right)-A\left(treated\right)}{A\left(untreated\right)+A\left(treated\right)}$$

Toxicity and fecundity assays were carried out in 9 cm Ø Petri dishes filled with moistened filter paper. A *C. pepo* leaf was placed on filter paper with the lower surface facing upwards, and ten apterous adults of *A. gossypii* were transferred onto it. The leaf was sprayed with EO or HS before and after aphid transfer. Leaves of the same age and size sprayed with deionized water provided the control treatment. The filter paper was moistened from time to time to maintain leaf turgidity, and Petri dishes were sealed with parafilm. The mortality rate of the aphids was recorded 24, 48, 72 and 96 h after treatment. Aphids were considered dead when they did not respond to gentle prodding with a brush. During each data collection instance, aphid offspring were also counted and removed. Twenty replicates were performed per EO or HS treatment and water treatment. Data from test choices, toxicity and fecundity assays were analyzed with Chi-square test to assess statistically significant differences between each treatment and the untreated control. For the test choice (repellency) assay, pairwise Chi-square test comparisons among treatments were also performed. 

## 5. Conclusions

The composition of EOs was very complex, due to the presence of tens of molecules belonging to monoterpene hydrocarbons and oxygenated monoterpenes. On the other hand, in the HS organic phase, a main compound was recognizable. 

The results of experiments involving both plant treatments and insect assays indicated good performance in reducing virus titer and repelling the aphid vector. *M. suaveolens* HS also showed toxicity and offspring inhibition characteristics against *A. gossypii*. The measured PAL expression levels in treated plants also suggested a mechanism of action based on the stimulation of plant defense response through the phenylpropanoid pathway. In the frame of envisaged integrated pest management to reduce the use of synthetic pesticides, these treatments represent potential biopesticides for the concurrent control of ZYMV and its vector in *C. pepo* crops with a single substance. Further studies are necessary to achieve a formulation applicable for practical use in the greenhouse and possibly in the field.

## Figures and Tables

**Table 1:** Summary of chemical classes retrieved in EOs and HSs used in this study expressed as percentage of the total ion current (TIC) as measured using GC-MS. Values are expressed as means of three chromatographic replicate runs. MS EO = *M. suaveolens* essential oil; MS HS = *M. suaveolens* hydrosol; FV EO = *F. vulgare* essential oil; FV HS = *F. vulgare* hydrosol.

Class of Components	MS EO	MS HS	FV EO	FV HS
Monoterpene hydrocarbons	21.13	-	26.91	6.02
Oxygenated monoterpenes	17.93	16.09	33.19	76.36
Sesquiterpene hydrocarbons	2.51	-	0.12	-
Phenolic compounds	4.11	3.94	0.10	-
Alcohols	7.55	1.52	-	-
Esters	9.85	4.74	18.47	-
Aromatic compounds	1.21	-	11.93	-
Nitrogen compounds	-	68.46	1.14	-
Oxygenated heterobicyclic	0.87	-	-	-
Total identified	83.00	94.75	92.45	82.38

**Table 2:** Results of toxicity assay on *M. suaveolens* and *F. vulgare* essential oils and hydrosols against *A. gossypii* adults. Twenty replicates were carried out, and 10 adults were used per replicate (N = 200). MS EO = *M. suaveolens* essential oil; MS HS = *M. suaveolens* hydrosol; FV EO = *F. vulgare* essential oil; FV HS = *F. vulgare* hydrosol. Toxicity is expressed as adult mortality percentage for each treatment; Chi-square values and significance of differences reported are based on Chi-square tests for each treatment vs. untreated control. Significance is expressed as ** = *p* < 0.01; *** = *p* < 0.001; **** = *p* < 0.0001.

Time	MS EO		MS HS		FV EO		FV HS	
	Mortality%	Chi-Square	Mortality%	Chi-Square	Mortality%	Chi-Square	Mortality%	Chi-Square
24 h	4.0	1.72	58.6 ****	80.11	8.0	0.01	25.0 ***	13.36
48 h	5.2 **	10.76	62.1 ****	34.57	15.2	1.08	26.7	1.10
72 h	11.0 **	7.93	54.5 **	7.64	7.7 ***	10.94	50.6 **	9.74
96 h	16.0	0.05	40.0	3.61	27.8	2.02	48.1 ***	12.66

**Table 3:** Results of daily progeny production assessment of *A. gossypii* adults exposed to *M. suaveolens* and *F. vulgare* EOs and HSs. Twenty replicates were carried out, and 10 adults were used per replicate (N = 200). MS EO = *M. suaveolens* essential oil; MS HS = *M. suaveolens* hydrosol; FV EO = *F. vulgare* essential oil; FV HS = *F. vulgare* hydrosol. Progeny is expressed as the number of offspring per adult for each treatment; Chi-square values and significance of differences reported are based on Chi-square tests for each treatment vs. untreated control. Significance is expressed as ** = *p* < 0.01; **** = *p* < 0.0001.

Time	MS EO		MS HS		FV EO		FV HS	
	Progeny	Chi-Square	Progeny	Chi-Square	Progeny	Chi-Square	Progeny	Chi-Square
24 h	0.82	0.93	0.13 ****	66.10	0.55 **	7.45	1.03	0.02
48 h	1.09	0.07	0.32 ****	23.56	0.88	1.62	1.48	1.31
72 h	1.30	0.18	0.48 **	10.19	1.24	0.35	1.27	0.20
96 h	1.19	0.08	0.47	3.19	1.50	1.58	1.38	0.49

## Data Availability

Not applicable.

## Supplementary Materials

The following supporting information can be downloaded at: https://www.mdpi.com/article/10.3390/plants12051078/s1, Figure S1: Flow diagram illustrating all treatments to plants and downstream analyses. Table S1: Detailed chemical composition of EOs and HSs used in this study.

## References

[1] Baser K.H.C., Buchbauer G. (2015). Handbook of Essential Oils.

[2] Božović M., Navarra A., Garzoli S., Pepi F., Ragno R. (2017). Esential Oils Extraction: A 24-Hour Steam Distillation Systematic Methodology. Nat. Prod. Res..

[3] Barton P., Hughes R.E., Hussein M.M. (1992). Supercritical Carbon Dioxide Extraction of Peppermint and Spearmint. J. Supercrit. Fluids.

[4] Bakkali F., Averbeck S., Averbeck D., Idaomar M. (2008). Biological Effects of Essential Oils—A Review. Food Chem. Toxicol..

[5] Božović M., Garzoli S., Baldisserotto A., Romagnoli C., Pepi F., Cesa S., Vertuani S., Manfredini S., Ragno R. (2018). Melissa Officinalis L. Subsp. Altissima (Sibth. & Sm.) Arcang. Essential Oil: Chemical Composition and Preliminary Antimicrobial Investigation of Samples Obtained at Different Harvesting Periods and by Fractionated Extractions. Ind. Crops Prod..

[6] Sabatino M., Fabiani M., Božović M., Garzoli S., Antonini L., Marcocci M.E., Palamara A.T., de Chiara G., Ragno R. (2020). Experimental Data Based Machine Learning Classification Models with Predictive Ability to Select in Vitro Active Antiviral and Non-Toxic Essential Oils. Molecules.

[7] Papa R., Garzoli S., Vrenna G., Sabatino M., Sapienza F., Relucenti M., Donfrancesco O., Fiscarelli E., Artini M., Selan L. (2020). Essential Oils Biofilm Modulation Activity, Chemical and Machine Learning Analysis—Application on Staphylococcus Aureus Isolates from Cystic Fibrosis Patients. Int. J. Mol. Sci..

[8] Barragán-Ocaña A., Silva-Borjas P., Olmos-Peña S. (2022). Scientific and Technological Trajectories for Sustainable Agricultural Solutions. Biopesticides.

[9] Labadie C., Cerutti C., Carlin F. (2016). Fate and Control of Pathogenic and Spoilage Micro-Organisms in Orange Blossom (*Citrus Aurantium)* and Rose Flower (*Rosa Centifolia)* Hydrosols. J. Appl. Microbiol..

[10] Xie L.H., Lin Q.Y., Wu Z.J. (2009). Plant. Virus: Virology and Molecular Biology.

[11] Rubio L., Galipienso L., Ferriol I. (2020). Detection of Plant Viruses and Disease Management: Relevance of Genetic Diversity and Evolution. Front. Plant Sci..

[12] Golino D.A., Fuchs M., Rwahnih M.A., Farrar K., Schmidt A., Martelli G.P., Meng B., Martelli G.P., Golino D.A., Fuchs M. (2017). Regulatory Aspects of Grape Viruses and Virus Diseases: Certification, Quarantine, and Harmonization. Grapevine Viruses: Molecular Biology, Diagnostics and Management.

[13] Fereres A., Raccah B. (2015). Plant Virus Transmission by Insects.

[14] Bishop C.D. (1995). Antiviral Activity of the Essential Oil of *Melaleuca Alternifolia* (Maiden Amp; Betche) Cheel (Tea Tree) Against Tobacco Mosaic Virus. J. Essent. Oil Res..

[15] Dunkić V., Bezić N., Vuko E., Cukrov D. (2010). Antiphytoviral Activity of Satureja Montana L. Ssp. Variegata (Host) P. W. Ball Essential Oil and Phenol Compounds on CMV and TMV. Molecules.

[16] Lu M. (2013). In Vitro and In Vivo Anti-Tobacco Mosaic Virus Activities of Essential Oils and Individual Compounds. J. Microbiol. Biotechnol..

[17] Bezić N., Vuko E., Dunkić V., Ruščić M., Blažević I., Burčul F. (2011). Antiphytoviral Activity of Sesquiterpene-Rich Essential Oils from Four Croatian Teucrium Species. Molecules.

[18] Abdel-Shafi S. (2013). Preliminary Studies on Antibacterial and Antiviral Activities of Five Medicinal Plants. J. Plant Pathol. Microbiol..

[19] Vuko E., Rusak G., Dunkić V., Kremer D., Kosalec I., Rađa B., Bezić N. (2019). Inhibition of Satellite RNA Associated Cucumber Mosaic Virus Infection by Essential Oil of Micromeria Croatica (Pers.) Schott. Molecules.

[20] Taglienti A., Donati L., Ferretti L., Tomassoli L., Sapienza F., Sabatino M., di Massimo G., Fiorentino S., Vecchiarelli V., Nota P. (2022). In Vivo Antiphytoviral Activity of Essential Oils and Hydrosols From Origanum Vulgare, Thymus Vulgaris, and Rosmarinus Officinalis to Control Zucchini Yellow Mosaic Virus and Tomato Leaf Curl New Delhi Virus in *Cucurbita pepo* L.. Front. Microbiol..

[21] D’Amato S., Serio A., López C.C., Paparella A. (2018). Hydrosols: Biological Activity and Potential as Antimicrobials for Food Applications. Food Control.

[22] Saǧdıç O., Özcan M. (2003). Antibacterial Activity of Turkish Spice Hydrosols. Food Control.

[23] Tornuk F., Cankurt H., Ozturk I., Sagdic O., Bayram O., Yetim H. (2011). Efficacy of Various Plant Hydrosols as Natural Food Sanitizers in Reducing Escherichia Coli O157:H7 and Salmonella Typhimurium on Fresh Cut Carrots and Apples. Int. J. Food Microbiol..

[24] Ozturk I., Tornuk F., Caliskan-Aydogan O., Durak M.Z., Sagdic O. (2016). Decontamination of Iceberg Lettuce by Some Plant Hydrosols. LWT.

[25] Li Y., Xu X., Wu Y., Zhuang J., Zhang X., Zhang H., Lei B., Hu C., Liu Y. (2020). A Review on the Effects of Carbon Dots in Plant Systems. Mater. Chem. Front..

[26] Servin A., Elmer W., Mukherjee A., de la Torre-Roche R., Hamdi H., White J.C., Bindraban P., Dimkpa C. (2015). A Review of the Use of Engineered Nanomaterials to Suppress Plant Disease and Enhance Crop Yield. J. Nanopart. Res..

[27] Somala N., Laosinwattana C., Teerarak M. (2022). Formulation Process, Physical Stability and Herbicidal Activities of Cymbopogon Nardus Essential Oil-Based Nanoemulsion. Sci. Rep..

[28] Abdollahdokht D., Gao Y., Faramarz S., Poustforoosh A., Abbasi M., Asadikaram G., Nematollahi M.H. (2022). Conventional Agrochemicals towards Nano-Biopesticides: An Overview on Recent Advances. Chem. Biol. Technol. Agric..

[29] Kumar A., Choudhary A., Kaur H., Mehta S., Husen A. (2021). Smart Nanomaterial and Nanocomposite with Advanced Agrochemical Activities. Nanoscale Res. Lett..

[30] Nair A., Mallya R., Suvarna V., Khan T.A., Momin M., Omri A. (2022). Nanoparticles—Attractive Carriers of Antimicrobial Essential Oils. Antibiotics.

[31] Nerio L.S., Olivero-Verbel J., Stashenko E. (2010). Repellent Activity of Essential Oils: A Review. Bioresour. Technol..

[32] Khursheed A., Rather M.A., Jain V., Wani A.R., Rasool S., Nazir R., Malik N.A., Majid S.A. (2022). Plant Based Natural Products as Potential Ecofriendly and Safer Biopesticides: A Comprehensive Overview of Their Advantages over Conventional Pesticides, Limitations and Regulatory Aspects. Microb. Pathog..

[33] Traka C.K., Petrakis E.A., Kimbaris A.C., Polissiou M.G., Perdikis D.C. (2018). Effects of Ocimum Basilicumand Ruta Chalepensis Hydrosols on Aphis Gossypii and Tetranychus Urticae. J. Appl. Entomol..

[34] Petrakis E.A., Kimbaris A.C., Lykouressis D.P., Polissiou M.G., Perdikis D.C. (2015). Hydrosols Evaluation in Pest Control: Insecticidal and Settling Inhibition Potential against Myzus Persicae (Sulzer). J. Appl. Entomol..

[35] Zekri N., Handaq N., el Caidi A., Zair T., Alaoui El Belghiti M. (2016). Insecticidal Effect of Mentha Pulegium L. and Mentha Suaveolens Ehrh. Hydrosols against a Pest of Citrus, Toxoptera Aurantii (Aphididae). Res. Chem. Intermed..

[36] Božović M., Pirolli A., Ragno R. (2015). Mentha Suaveolens Ehrh. (Lamiaceae) Essential Oil and Its Main Constituent Piperitenone Oxide: Biological Activities and Chemistry. Molecules.

[37] Ruberto G., Baratta M.T., Deans S.G., Dorman H.J.D. (2000). Antioxidant and Antimicrobial Activity of Foeniculum Vulgare and Crithmum Maritimum Essential Oils. Planta Med..

[38] lo Cantore P., Iacobellis N.S., de Marco A., Capasso F., Senatore F. (2004). Antibacterial Activity of Coriandrum Sativum L. and Foeniculum Vulgare Miller Var. Vulgare (Miller) Essential Oils. J. Agric. Food Chem..

[39] Kalleli F., Abid G., ben Salem I., Boughalleb-M’hamdi N., M’hamdi M. (2020). Essential Oil from Fennel Seeds (Foeniculum Vulgare) Reduces Fusarium Wilt of Tomato (Solanum Lycopersicon). Phytopathol. Mediterr..

[40] Pavela R. (2018). Essential Oils from Foeniculum Vulgare Miller as a Safe Environmental Insecticide against the Aphid Myzus Persicae Sulzer. Environ. Sci. Pollut. Res..

[41] Gal-On A. (2007). Zucchini Yellow Mosaic Virus: Insect Transmission and Pathogenicity? The Tails of Two Proteins. Mol. Plant Pathol..

[42] Walkey D.G.A., Lecoq H., Collier R., Dobson S. (1992). Studies on the Control of Zucchini Yellow Mosaic Virus in Courgettes by Mild Strain Protection. Plant Pathol..

[43] Desbiez C., Lecoq H. (1997). Zucchini Yellow Mosaic Virus. Plant Pathol..

[44] Blackman R.L., Eastop V.F. (2000). Aphids on the World’s Crops: An Identification and Information Guide.

[45] Shi X., Jiang L., Wang H., Qiao K., Wang D., Wang K. (2011). Toxicities and Sublethal Effects of Seven Neonicotinoid Insecticides on Survival, Growth and Reproduction of Imidacloprid-Resistant Cotton Aphid, Aphis Gossypii. Pest. Manag. Sci..

[46] Kim D.S., Hwang B.K. (2014). An Important Role of the Pepper Phenylalanine Ammonia-Lyase Gene (PAL1) in Salicylic Acid-Dependent Signalling of the Defence Response to Microbial Pathogens. J. Exp. Bot..

[47] Abdelkhalek A., Dessoki E.S., Hafez E. (2018). Polyphenolic Genes Expression Pattern and Their Role in Viral Resistance in Tomato Plant Infected with Tobacco Mosaic Virus. Biosci. Res..

[48] Zhao L., Feng C., Wu K., Chen W., Chen Y., Hao X., Wu Y. (2017). Advances and Prospects in Biogenic Substances against Plant Virus: A Review. Pestic. Biochem. Physiol..

[49] Raveau R., Fontaine J., Lounès-Hadj Sahraoui A. (2020). Essential Oils as Potential Alternative Biocontrol Products against Plant Pathogens and Weeds: A Review. Foods.

[50] Isman M.B. (2020). Bioinsecticides Based on Plant Essential Oils: A Short Overview. Z. Für. Nat. C.

[51] Barra A. (2009). Factors Affecting Chemical Variability of Essential Oils: A Review of Recent Developments. Nat. Prod. Commun..

[52] Sutour S., Bradesi P., Casanova J., Tomi F. (2010). Composition and Chemical Variability of Mentha Suaveolens Ssp. Suaveolens and M. Suaveolens Ssp. Insularis from Corsica. Chem. Biodivers..

[53] Lorenzo D., Paz D., Dellacassa E., Davies P., Vila R., Cañigueral S. (2002). Essential Oils of Mentha Pulegium and Mentha Rotundifolia from Uruguay. Braz. Arch. Biol. Technol..

[54] Anwar F., Ali M., Hussain A.I., Shahid M. (2009). Antioxidant and Antimicrobial Activities of Essential Oil and Extracts of Fennel (Foeniculum Vulgare Mill.) Seeds from Pakistan. Flavour. Fragr. J..

[55] Civitelli L., Panella S., Marcocci M.E., de Petris A., Garzoli S., Pepi F., Vavala E., Ragno R., Nencioni L., Palamara A.T. (2014). In Vitro Inhibition of Herpes Simplex Virus Type 1 Replication by Mentha Suaveolens Essential Oil and Its Main Component Piperitenone Oxide. Phytomedicine.

[56] Gulfraz M., Mehmood S., Minhas N., Jabeen N., Kausar R., Jabeen K., Arshad G. (2008). Composition and Antimicrobial Properties of Essential Oil of Foeniculum Vulgare. Afr. J. Biotechnol..

[57] Šilha D., Švarcová K., Bajer T., Královec K., Tesařová E., Moučková K., Pejchalová M., Bajerová P. (2020). Chemical Composition of Natural Hydrolates and Their Antimicrobial Activity on Arcobacter-Like Cells in Comparison with Other Microorganisms. Molecules.

[58] la Camera S., Gouzerh G., Dhondt S., Hoffmann L., Fritig B., Legrand M., Heitz T. (2004). Metabolic Reprogramming in Plant Innate Immunity: The Contributions of Phenylpropanoid and Oxylipin Pathways. Immunol. Rev..

[59] Gutha L.R., Casassa L.F., Harbertson J.F., Naidu R.A. (2010). Modulation of Flavonoid Biosynthetic Pathway Genes and Anthocyanins Due to Virus Infection in Grapevine (Vitis Vinifera L.) Leaves. BMC Plant Biol..

[60] Chen C., Cai N., Chen J., Wan C. (2019). Clove Essential Oil as an Alternative Approach to Control Postharvest Blue Mold Caused by Penicillium Italicum in Citrus Fruit. Biomolecules.

[61] Politi M., Menghini L., Conti B., Bedini S., Farina P., Cioni P.L., Braca A., de Leo M. (2020). Reconsidering Hydrosols as Main Products of Aromatic Plants Manufactory: The Lavandin (Lavandula × Intermedia) Case Study in Tuscany. Molecules.

[62] Sayed S., Soliman M.M., Al-Otaibi S., Hassan M.M., Elarrnaouty S.-A., Abozeid S.M., El-Shehawi A.M. (2022). Toxicity, Deterrent and Repellent Activities of Four Essential Oils on Aphis Punicae (Hemiptera: Aphididae). Plants.

[63] Andrade L.H.d., Oliveira J.V.d., Lima I.M.d.M., Santana M.F.d., Breda M.O. (2013). Efeito Repelente de Azadiractina e Óleos Essenciais Sobre Aphis Gossypii Glover (Hemiptera: Aphididae) Em Algodoeiro. Rev. Ciência Agronômica.

[64] Benddine H., Zaid R., Babaali D., Daoudi-Hacini S. (2022). Biological Activity of Essential Oils of Myrtus Communis (Myrtaceae, Family) and Foeniculum Vulgare (Apiaceae, Family) on Open Fields Conditions against Corn Aphids Rhopalosiphum Maidis (Fitch, 1856) in Western Algeria. J. Saudi Soc. Agric. Sci..

[65] Digilio M.C., Mancini E., Voto E., de Feo V. (2008). Insecticide Activity of Mediterranean Essential Oils. J. Plant Interact..

[66] Garzoli S., Pirolli A., Vavala E., di Sotto A., Sartorelli G., Božović M., Angiolella L., Mazzanti G., Pepi F., Ragno R. (2015). Multidisciplinary Approach to Determine the Optimal Time and Period for Extracting the Essential Oil from Mentha Suaveolens Ehrh. Molecules.

[67] Tsugawa H., Cajka T., Kind T., Ma Y., Higgins B., Ikeda K., Kanazawa M., VanderGheynst J., Fiehn O., Arita M. (2015). MS-DIAL: Data-Independent MS/MS Deconvolution for Comprehensive Metabolome Analysis. Nat. Methods.

[68] Misra B. Steps for Building an Open Source EI-MS Mass Spectral Library for GC-MS -Based Metabolomics. https://www.protocols.io/view/steps-for-building-an-open-source-ei-ms-mass-spect-eq2ly33rqgx9/v1.

[69] Sumner L.W., Amberg A., Barrett D., Beale M.H., Beger R., Daykin C.A., Fan T.W.-M., Fiehn O., Goodacre R., Griffin J.L. (2007). Proposed Minimum Reporting Standards for Chemical Analysis. Metabolomics.

[70] Manglli A., Bertin S., Tomassoli L. Preliminary Analysis of ZYMV and WMV Interaction in Mixed Infection by ΔΔCt Rt-QPCR. Proceedings of the International Advances in Plant Virology.

[71] Zhang S., Liu J., Xu B., Zhou J. (2021). Differential Responses of Cucurbita Pepo to Podosphaera Xanthii Reveal the Mechanism of Powdery Mildew Disease Resistance in Pumpkin. Front. Plant Sci..

[72] Livak K.J., Schmittgen T.D. (2001). Analysis of Relative Gene Expression Data Using Real-Time Quantitative PCR and the 2−ΔΔCT Method. Methods.

[73] Obrero Á., Die J.v., Román B., Gómez P., Nadal S., González-Verdejo C.I. (2011). Selection of Reference Genes for Gene Expression Studies in Zucchini (Cucurbita Pepo) Using QPCR. J. Agric. Food Chem..

