# Supplementary material for: In Vivo Antiphytoviral and Aphid Repellency Activity of Essential Oils and Hydrosols from Mentha suaveolens and Foeniculum vulgare to Control Zucchini Yellow Mosaic Virus and Its Vector Aphis gossypii
Source: Plants (Basel). 2023 Feb 28;12(5):1078. doi: 10.3390/plants12051078 (PMC10005592; doi:10.3390/plants12051078)
Supplement: Supplementary file 1 [file plants-12-01078-s001.zip › plants-2222283-supplementary.pdf]

In vivo antiphytoviral and aphid repellency activity of essential oils and hydrosols from *Foeniculum vulgare* and *Mentha suaveolens* to control zucchini yellow mosaic virus and its vector *Aphis gossypii*

Anna Taglienti [1]*, Livia Donati [1], Immacolata Dragone [1], Luca Ferretti [1], Andrea Gentili [1], Fabrizio Araniti [2], Filippo Sapienza [3], Roberta Astolfi [3], Simona Fiorentino [4], Valerio Vecchiarelli [4], Claudia Papalini [5], Rino Ragno [3] and Sabrina Bertin [1]

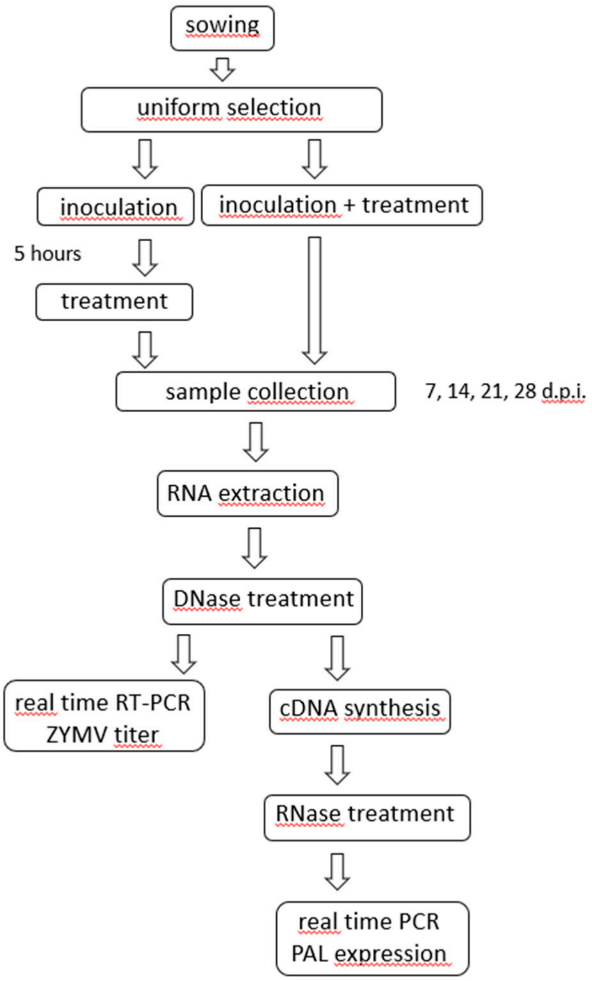

Figure S1. Flow diagram illustrating all treatments to plants and downstream analyses.

Table S1. Detailed chemical composition expressed as percentage of the Total Ion Current - TIC of EOs and HSs used in this study, as measured by GC-MS. Only compounds with relative abundance above 0.1% were reported. Retention index (RI) was calculated based on an alkane standard mixture (C8-C30), and values are expressed as a mean of three chromatographic replicate runs. MS EO = *M. suaveolens* essential oil, MS HS = *M. suaveolens* hydrosol, FV EO = *F. vulgare essential oil*, FV HS = *F. vulgare* hydrosol.

Component	Mean RI	MS EO	MS HS	FV EO	FV HS
Monoterpene hydrocarbons		*21.13*	-	*26.91*	*6.02*
α-thujene	927.71	0.10	-		-
α-pinene	934.90	3.11	-	4.14	1.17
camphene	951.47	-	-	0.19	-
β-thujene	975.98	1.52	-	0.17	1.27
β-pinene	979.80	4.71	-	1.78	-
β-myrcene	991.67	3.05	-	2.63	0.72
α-phellandrene	1009.07	-	-	3.99	-
α-terpinene	1010.96	-	-	0.25	-
o-cymene	1026.91	0.67	-	7.87	0.66
limonene	1031.34	-	-	5.75	2.21
menthene	1032.33	7.28	-	-	-
γ-terpinene	1046.00	0.14	-	0.14	-
3,7-dimethyl-1,3,6-octatriene	1048.60	0.14	-	-	-
α-terpinolene	1086.83	0.11	-	-	-
2-methylindan	1091.85	0.30	-	-	
Oxygenated monoterpenes		*17.93*	*16.09*	*33.19*	*76.36*
eucalyptol	1033.36	0.23	9.50	0.22	0.43
fenchone	1090.82	-	-	16.63	75.93
linalool	1098.85	-	0.38	-	-
camphor	1147.34	-	0.12	0.63	-
borneol	1172.06	-	1.12	0.11	-
terpinen-4-ol	1180.61	-	1.28	0.11	-
a-terpineol	1181.31	-	1.65	1.08	-
3-decanone	1183.81	0.17	-	-	-
p-cymen-8-ol	1187.56	1.23	1.03	-	-
myrtenol	1196.81	0.42	-		-
estragole	1200.04	-	-	2.66	-
carveol	1204.85	-	-	0.32	-
4,7-dimethylbenzofuran	1215.84	0.29	-	-	-
1-(2,4-dimethylphenyl)-ethanone	1217.77	0.88	-	-	-
(S)-carvone	1245.53	0.12	-	-	-
(S)-phellandral	1260.79	0.27	-	-	-
isopiperitenone	1270.45	0.85	1.01	-	-
anethol	1276.34	-	-	10.84	-
piperitenone	1339.86	0.82	-	-	-
durenol	1360.57	0.59	-	0.59	-
isomintlactone	1371.90	7.72	-	-	-
4-methoxyphenylacetone	1384.70	0.12	-	-	-
cis-isoeugenol	1395.34	0.39	-	-	-
nepetalactone	1398.86	3.83	-	-	-
Sesquiterpene hydrocarbons		*2.51*	-	*0.12*	-
copaene	1378.01	0.11	-	-	-
β-bourbonene	1385.72	0.37	-	-	-
cis-β-elemene	1391.39	0.26	-	-	-
α-bergamotene	1434.27	-	-	0.117	-
cis-murola-4(15),5-diene	1463.18	1.77	-	-	-
Phenolic compounds		*4.11*	*3.94*	*0.10*	-
sesamol	1286.67	0.24	-	-	-
thymol	1295.97	3.87	3.94	0.10	-
Alcohols		*7.55*	*1.52*	-	-
1-octen-3-ol	980.56	6.39	0.45	-	-

3-octanol	998.16	0.58	1.07	-	-
1-nonen-3-ol	1083.10	0.58		-	-
Esters		*9.85*	*4.74*	*18.47*	-
isobutyl isovalerate	1005.34	0.13	-	-	-
1-octen-3-yl-acetate	1110.30	2.31	-	-	-
3-octanol acetate	1122.11	0.25	-	-	-
3-hexenyl valerate	1238.15	0.31	-	-	-
pentanedioic acid, (p-t-butylphenyl)ester)	1297.67	-	-	18.47	-
geranyl acetate	1371.17	6.84	-	-	-
cytronellyl acetate	1357.88	-	4.74	-	-
Aromatic compounds		*1.21*	-	*11.93*	-
veratrole	1135.85	1.21	-	-	-
4-methoxybenzaldehyde	1260.79	-	-	4.52	-
1,2-dimethoxy-4-vinylbenzene	1381.03	-	-	1.65	-
4-methoxyphenylacetone	1383.92	-	-	5.76	-
Nitrogen compounds		-	*68.46*	*1.14*	-
N,N-Diethyl-m-toluidine	1292.31	-	-	1.14	-
decanenitrile	1293.54	-	68.46	-	-
Oxygenated heterobiyclic		*0.87*	-	-	-
dihydroedulan II	1294.32	0.87	-	-	-
Total identified		83.00	94.75	92.45	82.38
